# A Review of the Impact of Dietary Intakes in Human Pregnancy on Infant Birthweight

**DOI:** 10.3390/nu7010153

**Published:** 2014-12-29

**Authors:** Jessica A. Grieger, Vicki L. Clifton

**Affiliations:** Robinson Research Institute, School of Paediatrics and Reproductive Health, Adelaide University, Lyell McEwin Hospital, Haydown Road, Elizabeth Vale, SA 5112, Australia; E-Mail: vicki.clifton@adelaide.edu.au

**Keywords:** maternal nutrition, birthweight, undernutrition, overweight, nutrients, dietary patterns

## Abstract

Studies assessing maternal dietary intakes and the relationship with birthweight are inconsistent, thus attempting to draw inferences on the role of maternal nutrition in determining the fetal growth trajectory is difficult. The aim of this review is to provide updated evidence from epidemiological and randomized controlled trials on the impact of dietary and supplemental intakes of omega-3 long-chain polyunsaturated fatty acids, zinc, folate, iron, calcium, and vitamin D, as well as dietary patterns, on infant birthweight. A comprehensive review of the literature was undertaken via the electronic databases Pubmed, Cochrane Library, and Medline. Included articles were those published in English, in scholarly journals, and which provided information about diet and nutrition during pregnancy and infant birthweight. There is insufficient evidence for omega-3 fatty acid supplements’ ability to reduce risk of low birthweight (LBW), and more robust evidence from studies supplementing with zinc, calcium, and/or vitamin D needs to be established. Iron supplementation appears to increase birthweight, particularly when there are increases in maternal hemoglobin concentrations in the third trimester. There is limited evidence supporting the use of folic acid supplements to reduce the risk for LBW; however, supplementation may increase birthweight by ~130 g. Consumption of whole foods such as fruit, vegetables, low-fat dairy, and lean meats throughout pregnancy appears beneficial for appropriate birthweight. Intervention studies with an understanding of optimal dietary patterns may provide promising results for both maternal and perinatal health. Outcomes from these studies will help determine what sort of dietary advice could be promoted to women during pregnancy in order to promote the best health for themselves and their baby.

## 1. Introduction

Optimal nutrition supply to the developing fetus is paramount in achieving appropriate fetal growth and development. During pregnancy, dietary energy and nutrient requirements are generally increased to support increased maternal metabolism, blood volume and red cell mass expansion, and the delivery of nutrients to the fetus [1,2]. In a recent systematic review and meta-analysis of 90 dietary studies among pregnant women in developed countries (*n* = 126,242), compared to dietary recommendations in the specific countries, energy and fiber intakes were generally lower, total fat and saturated fat intakes were higher, and carbohydrate intake was borderline or lower than recommendations [3]. Key nutrients including folate, iron, zinc, calcium, vitamin D, and essential fatty acids function to promote red blood cell production, enzyme activity, bone development, and brain development. Current evidence indicates, however, that micronutrient intake during pregnancy is less than optimal [4]. This is of concern given the current consensus that maternal nutrition is relevant to both the short- and long-term health of the infant.

Human studies assessing maternal dietary intakes on birth outcomes date back to the early 1940s, when the appropriate energy and protein intakes to achieve a full-term infant (≥2500 g) were uncertain [5,6,7,8]. However, the available evidence at the time suggested that mothers with insufficient weight gain during pregnancy had higher rates of premature birth than mothers with appropriate pregnancy weight gain [9,10]. Undernutrition, then, was seen to markedly affect pregnancy outcome, potentially through inadequate energy availability to the developing fetus. Comparatively, the recent surge in the prevalence of overweight and obesity worldwide has been identified as another significant complication of pregnancy [11,12,13], potentially via fetal exposure to excess energy availability. The long-term detrimental influence of maternal obesity and excess maternal nutrition on the risk of disease in childhood and beyond has been described [14,15,16].

Unfortunately, there has been no single comprehensive review examining infant birthweight related to important maternal nutrients that may play a role in fetal birthweight. Therefore, this review will provide updated information from epidemiological studies and the latest evidence from systematic reviews/meta-analyses on the impact of long chain omega 3 polyunsaturated fatty acids (LC *n3* PUFA), zinc, iron, folate, calcium, and vitamin D on fetal birthweight. These nutrients were chosen due to their nutritional importance in pregnancy and the potential mechanisms relevant to birthweight. A distinction will be made between studies conducted in developed and developing countries due to differences in dietary intakes and prevalence of infants with low birthweight (LBW). Maternal nutrient deficiencies can occur in women who are of normal weight, obese, or underweight, and thus the impact of nutrient deficiencies will be discussed in the context of pregnancy undernutrition and overnutrition. The impact of whole dietary patterns on birthweight will also be discussed in order to highlight diet quality as a global holistic marker of nutritional intake.

## 2. Methods

A comprehensive review of the literature was undertaken via the electronic databases PubMed, Cochrane Library, and Medline. The most recent systematic reviews and meta-analyses were used to capture information from epidemiological and RCTs, with any subsequent studies published after the systematic reviews also included. Reviews were initially searched for using the search keywords “omega 3 fatty acids birthweight small for gestational age pregnancy”; “zinc birthweight small for gestational age pregnancy”; “iron birthweight small for gestational age pregnancy”; “folate birthweight small for gestational age pregnancy”; “calcium birthweight small for gestational age pregnancy”; “vitamin D birthweight small for gestational age pregnancy”; and “diet birthweight small for gestational age pregnancy”. Articles were limited to human studies published in English, and articles were included where they provided information about diet and nutrition during pregnancy and the birthweight outcomes of the fetus. Outcomes focussed on low birth weight (LBW: <2500 g) and small for gestational age (SGA: <10th percentile for gestational age), but also included intrauterine growth restriction (IUGR: <3rd percentile for gestational age) and large for gestational age (LGA: >90th percentile for gestational age); and macrosomia (>4000 g at birth). Key mechanistic studies were included to highlight the importance of different nutrients during pregnancy and transfer through the placenta. Supporting animal studies were included where there was a lack of human studies on the effects of maternal under- and overnutrition.

## 3. Review

### 3.1. Undernutrition and Fetal Growth

The major determinant of intrauterine fetal growth is the placental supply of nutrients to the fetus, which is dependent upon placental size, morphology, and blood supply [17]. Several animal studies have shown direct relationships between placental size and birthweight [18,19]. Experimental restriction of placental growth [20,21,22], food restriction [23], and isocaloric low protein diets [24,25] resulted in reduced placental weight and altered placental efficiency, leading to reduced birthweight and IUGR. The timing of delivery of nutrients through the placenta is also important [26]. In pregnant sheep, severe undernutrition during the peri-conceptual period led to preterm delivery [17]; global undernutrition in early pregnancy reduced the placental:fetal weight ratio [27]; global undernutrition in early to mid-gestation increased placental size [28,29]; and global undernutrition in late gestation reduced fetal growth [30]. In rats, a low protein diet in early pregnancy increased placental growth [31], whereas in late pregnancy there was reduced fetal weight [32]. A recent review has neatly summarized the animal models, demonstrating how dietary manipulation impacts perinatal programming [33]. It can be summarized that following maternal reduced nutrition (*i.e.*, a low protein isocaloric diet, or global food restriction at 20%–70% calorie intake), offspring exhibited structural disorganization and impaired programming of the appetite-regulating system located in the hypothalamus. Due to changes in orexigenic pathways and increased white adipose tissue, the potential increased risk of obesity later in life is inevitable.

Maternal undernutrition during critical periods in humans may also influence fetal adipocyte metabolism and fat mass, leading to obesity in later life [34]. Thus, alterations in fetal nutritional supply can drive developmental adaptations influencing growth and metabolism. These alterations affect not only postnatal outcomes but also contribute to increased susceptibility to disease in later life.

In a systematic review and meta-analysis of 78 studies (*n* = 1,025,794 women), risk of preterm birth was increased in the cohort studies of underweight women by 29%, and a 64% increased risk for LBW [35]. Low birthweight is a proxy for maternal undernutrition [36]. It is associated with reduced fat mass and lean mass [37,38], poor cognition in childhood [39], and increased risk for type 2 diabetes [40] and high blood pressure [41,42] in adulthood. These studies support a link between maternal nutrition, infant birthweight, and the short- and long-term consequences. However, there are limited epidemiological studies suggesting the optimal maternal dietary intakes for optimal infant birthweight.

### 3.2. Epidemiological Studies and Randomized Controlled Trials Assessing Birthweight

#### 3.2.1. Long chain omega-3 Polyunsaturated Fatty Acids

Fatty acids are among the essential nutrients for intrauterine growth [43]. Among the long chain omega-3 polyunsaturated fatty acids (LC *n3* PUFA), docosahexanoic acid (DHA) plays a critical role in fetal growth and central nervous system development. It has been estimated that the average complete DHA accretion rate *in utero* is 43 mg/kg/day [44]. As the fetus does not accumulate appreciable amounts of fat until the last trimester of gestation, if adequate DHA is not available during this critical window of development, the deleterious effect on the brain dopaminergic and serotonergic systems is irreversible [45]. There has been much recent investigation into the importance of LC *n3* PUFA in pregnancy [46,47,48] and associations between LC *n3* PUFA intakes and birth outcomes is emerging.

The most recent review that included 14 epidemiological studies assessing fish/seafood intake on birth outcomes revealed a positive association in 6 of the studies; 4 studies showed an inverse association, and the remaining studies revealed no association [49]. Overall in these studies, daily intake of fish ranged from 3.4 g to 47 g, or 8–12 fish meals per month. It is noted that 11 of the studies collected dietary data during pregnancy, while 3 studies collected dietary information following delivery [49].

There is emerging research, particularly in developed countries on *n3* fatty acid supplementation and birthweight. The most recent meta-analysis of 15 RCTs reported that birthweight was slightly higher among infants born to women in the LC *n*-3 PUFA supplemented group compared to placebo (RR 42.2; 95% CI: 14.8, 69.7); however, risk of LBW was not significantly different (RR 0.92; 95% CI: 0.83, 1.02) [49] (Table 1). In a further phase 3 double blind RCT with smaller sample size (*n* = 303), supplementation with 600 mg/day DHA from <20 week gestation to birth led to a 172 g greater birthweight [50]. Interestingly, it was reported that only 78% of the capsules were consumed, thus mean daily intakes of DHA was lower than anticipated (469 mg/day). The conflicting evidence of an increase in birthweight with LC *n3* PUFA supplementation and the lack of studies in developing countries suggest the need for further, high-quality studies to be replicated and confirmed. Moreover, the clinical significance of an increase in birthweight of 42 g and 172 g, identified by Imhoff-Kunsch *et al.* [49] and Carlson *et al.* [50], is unclear, particularly as supplementation only marginally affected LBW.

#### 3.2.2. Zinc

Zinc is essential for normal fetal growth and development [51]. In the USA, dietary zinc intake is recommended to increase from 8 mg/day in non-pregnant women to 11 mg/day during gestation [52]. Most of the zinc gained is deposited in the fetus and in the uterine muscle. Several studies have identified that maternal zinc absorption is not significantly different throughout gestation or compared to the non-pregnant state [51,53,54], but does appear to increase during lactation [51,55]. Potential mechanisms for adjustment of zinc metabolism may include reduced endogenous gastrointestinal zinc excretion [51,56] or the release of maternal tissue zinc [57].

Zinc deficiency is thought to influence embryonic and fetal development through reduced cell proliferation, reduced protein synthesis, or reductions in rates of tubulin polymerization, rather than increased rates of cellular oxidative damage, increased rates of apoptosis, or reduced binding of hormones and transcription factors dependent on zinc-finger regions [58,59]. Studies in animals have identified that zinc deficiency is teratogenic, such that it can interfere with normal embryonic development. For instance, offspring of mother hens with zinc deficiency were born with several skeletal defects and abnormalities of the brain, with many deaths occurring within 4 days [60], and when rats were fed a zinc-deficient diet during pregnancy, fewer offspring were born and many were growth-retarded, with multiple anomalies [61]. The consideration that zinc deficiency is a teratogenic risk in humans may be supported by the correlation of low plasma zinc concentrations in the first and third trimesters of pregnancy with respective increases in risk for malformations [62] and LBW [63]. Other adverse consequences of maternal zinc deficiency include increased maternal mortality, prolonged labor, prematurity [64], and adverse fetal development [65].

In a review of 46 studies conducted in developed and developing countries, 23 studies reported a positive relationship between maternal zinc concentrations and birthweight [64]. Since publication of that review, 3 studies have reported on zinc and birthweight, all in developing countries: 2 studies reported no relationship between low maternal zinc status and LBW infants [66,67], whereas among pregnant women from Tanzania, increased odds of LBW were observed with low maternal zinc concentrations [68]. Only a few studies assessing maternal dietary intakes of zinc were found, of which a study among women from New Jersey (*n* = 818) reported those with intakes of ≤6 mg/day had a two-fold increased risk of LBW compared to intakes >6.1 mg/day [69]; however, dietary intakes of zinc between 8 and 11 mg/day were not associated with LBW [70,71]. Currently there is insufficient evidence to presume that maternal zinc deficiency is teratogenic or that maternal zinc deficiency is related to neonatal birthweight. Further studies are required assessing mechanistic pathways between maternal zinc status and infant birthweight.

In the most recent Cochrane review of 20 RCTs, compared to placebo (or other micronutrient supplementation combinations), zinc supplementation (5–44 mg/day) had no effect on reducing risk for LBW or SGA [72] (Table 1). Similarly, in a systematic review and meta-analysis of 20 trials that was published in the same year, maternal zinc supplementation (between 5 and 50 mg/day) had no effect on LBW or SGA [73] (Table 1). In that review, however, only 6 trials were deemed to be of high quality, and the overall quality of evidence for LBW or SGA was very low. More robust evidence from supplementation studies needs to be established, and appropriate biomarkers to assess zinc status may need to be developed to support increasing dietary zinc intakes during pregnancy for infant birthweight.

#### 3.2.3. Iron

Dietary iron requirements during pregnancy increase due to the expansion of red cell mass to accommodate fetal and placental growth, and to allow for the blood loss that occurs at delivery. Increased dietary iron requirements are difficult to achieve through diet alone, and beginning pregnancy with already low intakes increases the likelihood of outcomes such as preterm birth and LBW [74,75]. Thus, the prevalence of iron deficiency (with and without anemia) is reported to increase during each trimester [76,77].

Only 1 study was located assessing dietary iron intakes on fetal body weight. In this study among 1274 pregnant women aged 18–45 years from the UK, it was found that for every 10 mg increase in dietary iron intake, fetal birthweight was predicted to increase by 70 g (95% CI: 10, 130; *p* = 0.02); however, when adjusting for maternal age, cotinine levels, alcohol intake, maternal weight, height, parity, ethnicity, gestational age, and fetal sex, the change of 34 g was not significant [78].

There is considerably more information on maternal iron status and birthweight. A recent review of 25 observational studies revealed anemia (defined as Hb < 110 g/L) during pregnancy increased the risk for LBW (OR 1.25; 95% CI: 1.08, 1.25) compared to non-anemic women (Hb ≥ 110 g/L); however, when including only the nine studies with adjusted estimates, this was no longer significant (aOR 1.13; 95% CI: 0.94, 1.35) [79]. Interestingly, there was a 30% increased risk for LBW when anemia occurred during the first two trimesters of pregnancy compared to the final trimester. Maternal anemia was not associated with birthweight or SGA, and there was no significant difference in birthweight between high- and low-income countries [79].

Some studies have also assessed ferritin concentration, which is an indicator of iron stores. Both low ferritin (<12 g/L, indicating depleted iron stores) and high ferritin (≥60 g/L) levels were significantly associated with lower birthweight (106 g and 123 g, respectively) compared with women with intermediate ferritin concentrations [74]. In a sample of 580 black women from Alabama, USA, plasma ferritin levels in the highest quartile at 19 weeks (95.8 µg/L), 26 weeks (55.4 µg/L), and 36 weeks (41.4 µg/L) were associated with a lower mean birthweight than those in the lower three quartiles, by 142–226 g [80], whereas infants of mothers with depleted iron stores (*n* = 51, serum ferritin <10.0 µg/L) had a lower birth weight by ~336 g compared to infants of mothers with adequate serum ferritin >20.0 µg/L (*n* = 20) [81]. A final study reported serum ferritin <12 µg/L was associated with 192 g lower birthweight [82].

**Table 1:** Summary of supplementation studies and relative risk for low birthweight.

Study Population	Supplementation Intervention	RR (95% CI)
*Long chain omega-3 polyunsaturated fatty acids*		
Meta-analysis of 15 RCTs [49]	80 mg/day–2.2 g/day (8 trials, *n* = 3247)	0.92 (0.83, 1.02)
*Zinc*		
Cochrane review of 20 RCTs (*n* > 15,000 women) [72]	5–44 mg/day (14 trials, *n* = 5643)	0.93 (0.78, 1.12)
	5–44 mg/day (8 trials, *n* = 4252)	1.02 (0.94, 1.11) ^a^
Systematic review and meta-analysis of 20 RCTs (*n* = 6209) [73]	15–50 mg/day (*n* = 11 trials, *n* = 937/5416 LBW babies)	1.06 (0.91, 1.23)
	25–45 mg/day (5 trials, *n* = 1155/3441 SGA babies)	1.03 (0.91, 1.23) ^a^
*Iron*		
Meta-analysis of 48 RCTs (*n* = 17,793) [79]	10–140 mg/day (*n* = 10 trials)	0.81 (0.71, 0.91)
		0.84 (0.66, 1.07) ^a^
*Folate*		
Cochrane review of 31 trials (*n* = 17,771) [83]	Dose range not reported (4 studies, *n* = 3113 on LBW)	0.83 (0.66, 1.04)
*Calcium*		
Cochrane review of 21 trials (*n* = 16,602) [84]	≥1000 mg/day (5 trials, *n* = 13,638, mainly calcium carbonate)	0.83 ( 0.63, 1.09)
	≥600 mg/day (5 trials, *n* = 1177, mainly calcium carbonate)	0.86 (0.61, 1.22) ^b^
*Vitamin D*		
Cochrane review of 6 trials on various maternal and infant outcomes (*n* = 623) [85]	1000 IU/day; 600,000 IU at month 7 and 8; 1 dose of 200,000 IU in third trimester (3 trials, *n* = 463)	0.48 (0.23, 1.01) ^b^

^a^ small for gestational age; ^b^ intrauterine growth restriction.

In the same review described above [79], a meta-analysis on 16 RCTs was carried out that assessed iron supplementation compared to control supplementation on birthweight (Table 1). Iron-only supplementation led to a mean difference in birthweight of 40.8 g (0.97 to 80.6) and reduced LBW (RR 0.81; 95% CI: 0.71, 0.91), but not SGA. Although duration of iron supplementation was not associated with birthweight, it was found that for every 10 mg increase in daily iron intake, birth weight increased by 15.1 (6.0 to 24.2) g, and for every 10 g/L increase in mean Hb concentration in the third trimester or at delivery, birthweight increased by 143 (95% CI: 68, 218) g [79].

Mechanisms linking iron status to birthweight are not established. An early study identified an inverse relationship between hemoglobin levels and placenta weight [86], in which further possible roles of the placenta in the regulation of iron transfer and the transport proteins involved have been implicated [87,88,89]. Additional mechanistic studies are needed to better understand the link between iron status and birthweight, as multiple studies have reported positive effects.

#### 3.2.4. Folate

Folate requirements are increased during pregnancy due to the demand of fetal growth [90]. It has long been established that folic acid at or around the time of conception reduces the risk for neural tube defects [91,92,93], and it is currently recommended that a 400 µg/day folic acid supplementation is taken three months before and early on in pregnancy [52,94,95]. However, the effect of routine folic acid supplementation on birth outcomes is not currently known.

Maternal folate status is largely determined by folate supplement use and though a number of other factors such as dietary folate intake and genetic variations in genes [90]. Red blood cell folate (RBC) is a biomarker of long-term folate status reflecting the previous 2–4 months, while serum and plasma folate represent short-term folate status and are therefore commonly used to reflect current folate supplement use [96].

No systematic reviews were located that reported on folate status and birth outcomes. However, in the most recent review of the literature assessing folate status and birth outcomes [97], maternal RBC folate status appeared to be associated with birthweight (*n* = 5 studies), and there was an increased risk for SGA with low or decreasing RBC folate (125–300 nmol/L) in two of four studies. However the majority of these studies were of poor quality, folate status was collected at different time points during gestation, and some of the studies were underpowered and thus were unable to identify a meaningful outcome. Comparatively, a number of high-quality studies were identified that assessed serum or plasma folate on birth outcomes. Only 2 of 15 studies reported significant associations between high or increasing serum or plasma folate during pregnancy and increased birthweight, while 1 study showed a significantly decreased risk of LBW with increasing folate levels in the second trimester. Folic acid supplement use was identified in 18 studies, of which 5 reported positive outcomes with supplement use in the second and third trimesters; and there was mixed evidence reporting folic acid supplement use and LBW or SGA. Although the majority of these studies were of high quality, some studies reported pre-conception folate intake and some RCTs were also summarized in the results. Thus, the ability to draw appropriate conclusions about the effect of dietary folate intake and folate status on birth outcomes is difficult. No further studies have been carried out since that review, further limiting the ability to understand the impact of maternal folate intake on birth outcomes, and at what time point during gestation folate should be measured.

In a Cochrane review assessing folic acid alone or with other micronutrients *versus* no folic acid (Table 1), the mean difference in birthweight was significantly greater in the folic acid supplement groups compared to no folic acid supplement (mean difference 135.75 g; 95% CI: 47.85, 223.68), but not LBW (RR 0.83; 95% CI: 0.66, 1.04; 4 studies, *n* = 3113 participants) [83]. In that review, it was revealed that supplementation typically took place from the 8th week of pregnancy until three days postpartum. The indication that most studies were also conducted 30–45 years ago suggests the need for current, well-designed clinical trials to assess the effectiveness of folic acid supplement use on birthweight parameters, given that current folic supplement use is recommended to assist other neonatal outcomes, namely neural tube defects.

#### 3.2.5. Calcium

Calcium absorption and urinary calcium excretion are approximately two-fold higher during pregnancy compared to pre-conception or after delivery [98,99,100]. These changes are evidenced at the end of the first trimester and available for the peak fetal demands in the third trimester. Calcium is transported across the placenta by an active transport process, being important in many developmental functions and critical for skeletal development [101]. There does not appear to be an additional need for calcium in pregnancy [102].

Few studies have reported on associations between calcium intake and birthweight. Studies in developing countries have reported higher calcium intakes (≥1200 mg/day *vs.* <800 mg/day) were associated with higher birthweight (~3400 g *vs.* 3000 g) [71], particularly when calcium intakes reached dietary recommendations [103,104]. It is to be noted, however, that birthweight of 3000 g with lower daily calcium intake of 800 mg is not considered an inappropriate birthweight.

In a recent Cochrane review of 21 trials, no effect of calcium supplementation was found for LBW or IUGR, despite all 5 trials providing high dose supplementation (≥1000 mg/day) [84] (Table 1). Comparatively, higher birthweights were apparent following calcium supplementation compared to placebo (mean difference 64.66 g; 95% CI: 15.75, 113.58) in 19 trials with 8287 women [84]; however, the clinical importance of an increase of 64 g is unknown.

#### 3.2.6. Vitamin D

The major circulating form of vitamin D is 25-hydroxyvitamin D (25(OH)D) and the most biological potent form is calcitriol (1,25-dihydroxyvitamin D_3_) (1,25(OH)_2_D_3_)). 25(OH)D readily crosses experimentally perfused placentas in humans [105,106] and rodents [107], such that cord blood 25(OH)D levels at term are typically three-quarters that of maternal values [108]. In comparison, 1,25(OH)_2_D_3_ does not appear to cross the placenta, with lower levels detected in cord blood [109]. Although there is no recommended increase in vitamin D intake during pregnancy [95,110], it is apparent that neonates born to vitamin D-deficient mothers will also be deficient [111,112]. There is mixed evidence regarding the effect of maternal 25(OH)D on bone health at birth; however, studies assessing skeletal health in childhood suggest 25(OH)D levels during pregnancy may have more impact at this later stage [113].

Currently, the estimated average requirement for calcium and vitamin D during pregnancy according to the Institute of Medicine (IOM) is 800 mg/day and 400 IU/day (10 µg/day), respectively [110]. The recommendation for calcium is similar to that reported by the National Health and Medical Research Council (NHMRC) in Australia (840 mg/day); however, the requirement for vitamin D is half (5 µg/day, 200 IU/day) [95].

The most recent meta-analysis revealed that 25(OH)D insufficiency (<37.5 nmol/L) during pregnancy was associated with increased risk for small SGA (OR 1.85; 95% CI 1.52, 2.26, 6 studies) compared to higher 25(OH)D levels [114]. Importantly, even adjusting for confounders such as 25(OH)D concentration cut-offs (<37.5 and <80 nmol/L), gestational age (<16 and >16 weeks), and study design (case-control and other), the association remained significant. In the same review that included 4 studies on LBW, mothers with 25(OH)D concentrations <37.5 nmol/L during pregnancy had infants with lower birth weight (random weighted mean difference −130.92; 95% CI 186.69, 75.14 g) compared to infants of mothers with levels greater than this [114].

A Cochrane review of 3 RCTs identified vitamin D treatment alone as borderline significant in reducing risk for SGA compared to no supplements/placebo (Table 1) [85]. Currently, there is insufficient evidence to promote the use of a vitamin D supplement (with or without calcium) during pregnancy to reduce the risk of LBW babies. The NHMRC in Australia recommends consuming 5 µg/day vitamin D [95] whereas the IOM advocates 10 µg/day for those in the USA or Canada [110]. In Australia, it has also been suggested that pregnant women who receive regular exposure to sunlight do not require supplementation; however, for those who have little access to sunlight, a vitamin D supplement of 10 µg/day would not be excessive [95]. Although various vitamin D supplements are currently on the market in doses ranging from 5 to10 µg, further studies are required to assess whether supplemental vitamin D can improve infant health outcomes.

### 3.3. Dietary Pattern Studies

Assessment of single nutrients on various study outcomes such as birthweight presents several caveats as a result of the highly interrelated nature of dietary exposures. Thus it is difficult to separate out the specific effects of nutrients or foods in relation to infant birthweight, disease risk, or other health outcomes. Studies assessing whole foods rather than specific nutrients may provide valuable information on optimal nutrition during pregnancy, and therefore dietary pattern analysis aims to overcome some of the difficulties that are often lost in nutrient-based analyses. Higher consumption of food groups within each identified dietary pattern indicates higher scores/adherence to this type of pattern.

Among women participating in the Auckland Birthweight Collaborative study (*n* = 1714), higher scores on the *traditional* food pattern (characterized by fruit, vegetables, yogurt, and lean meat) in early pregnancy was associated with reduced odds for SGA (OR 0.86; 95% CI: 0.75, 0.99) [115]. Similarly, compared to the *Western* dietary pattern (*n* = 7619) characterized by a high intake of high-fat dairy, refined grains, processed meat, beer, and sweets, 26%–32% reduced odds for SGA were found for both the *health conscious* dietary pattern (*n* = 7479), characterized by a higher intake of fruits, vegetables, poultry, and breakfast cereals, and for the *intermediate* dietary pattern (*n* = 29,514), characterized by low fat dairy and fruit but also including some red meat, dairy, and vegetables [116]. Among women from Spain, higher scores on the Alternate-Healthy Eating Index in the first trimester were associated with reduced risk of fetal growth restriction compared to women with the lowest score [117]. Among women participating in the Osaka Maternal and Child Health Study in Japan, those in the wheat products pattern (bread, confectionery, and soft drinks, *n* = 303) had infants with lower birthweight, and compared with women in the rice, fish, and vegetables pattern, women in the *wheat products* pattern had higher odds of an infant being born SGA (OR 5.2; 95% CI: 1.1, 24.4) [118].

In pregnant women from the Generation R study in the Netherlands, both low and medium adherence to the Mediterranean diet in the first trimester was associated with lower birthweight [119]. In two population-based cohort studies from Spain, higher intakes of fish, legumes, and dairy products were associated with higher infant birthweight and reduced risk for IUGR, while intake of cereal, fruits, and nuts had no relationship with birthweight [120]. Similarly, in women from the Danish National Birth Cohort (*n* = 35,530), higher intake of Mediterranean foods reduced the odds for preterm birth [121].

The first dietary pattern analysis to assess *pre-conception* diet on perinatal outcomes was carried out in a sample of 309 pregnant women from an area of low socioeconomic status in Australia [122]. The study revealed no association between any of the dietary patterns identified with LBW or SGA; however, there was an approximate 50% reduced risk for preterm delivery following the high protein and fruit pattern, and there was a 50% increased risk for preterm delivery following a high fat, sugar, and takeaway pattern [122].

There is emerging data on dietary pattern assessment and fetal growth. Generally, these studies were reported in large prospective cohorts, thus capturing a large sample size, which improves the generalizability to other populations. The single study that assessed pre-conception diet and perinatal outcomes warrants further investigation, as improving diet pre-conception presents an ideal opportunity to modify dietary intake prior to pregnancy in order to optimize birth outcomes. Nevertheless, these studies suggest that consumption of whole foods such as fruit, vegetables, low-fat dairy, and lean meats throughout pregnancy appears beneficial in reducing risk for LBW babies, but it is unclear if healthier food choices in a certain trimester are optimal. Assessing quantities of the different foods relevant to national food consumption guidelines would also be a valuable component to dietary pattern analyses.

### 3.4. Summary

According to the reviewed epidemiological studies, there does not appear to be any consistent or conclusive evidence regarding the optimal intake for maternal dietary LCPUFA, zinc, calcium, or vitamin D for optimal birthweight. Larger studies are needed that assess LCPUFA intakes and blood concentrations over the course of pregnancy, and to determine at what time point and at what level is most important in determining optimal birth outcomes. There is insufficient evidence to presume that maternal zinc deficiency is teratogenic or that maternal zinc deficiency is related to neonatal birthweight; therefore, further studies are required assessing mechanistic pathways between maternal zinc status and infant birthweight. Iron supplementation increases infant birthweight and the magnitude that was identified with higher Hb concentrations (*i.e.*, for each 10 g/L increase in mean Hb concentration in late pregnancy or at delivery, birthweight increased by 143 g), could be critical for the survival of neonates born with lower birthweight. There is limited evidence supporting the use of folic acid supplements to reduce the risk for LBW; however, supplementation may increase birthweight by ~130 g. Further trials assessing calcium supplementation need to be undertaken, particularly among women with low baseline calcium intakes, to support the few epidemiology studies identifying higher calcium intakes with higher birthweight. Serum 25(OH)D levels of at least 37.5 nmol/L appear optimal in reducing the risk for SGA and increasing birthweight; however, clarification as to what defines a normal 25(OH)D level is required, in addition to high-quality study designs. Currently there is not enough evidence to support the use of fish oil supplements to reduce the risk of LBW in low-risk or high-risk pregnancies, and more robust evidence from studies supplementing with zinc, calcium, and/or vitamin D needs to be established. Attempting to draw inferences on the role of maternal nutrition in determining the fetal growth trajectory is difficult. Dietary pattern studies appear to show positive associations between healthier dietary patterns and infant birthweight. In particular, foods such as lean meat, vegetables, fruit, whole grains, and low-fat dairy all contain beneficial nutrients such as protein, fiber, iron, zinc, calcium, and folate. Intervention studies assessing whole foods rather than specific nutrients may provide valuable information on optimal nutrition during pregnancy, and how certain dietary patterns improve maternal and perinatal outcomes. Outcomes from these studies will help determine what sort of dietary advice could be promoted to women during pregnancy in order to promote the best health for themselves and their babies.

### 3.5. Overnutrition and Fetal Growth

The global increase in overweight and obesity [11,12,13] indicates that more women entering pregnancy are overweight/obese [11,123]. Maternal BMI is a proxy measure of nutritional status [12,124,125,126,127,128,129]. Epidemiological studies have reported that maternal overweight (*i.e.*, BMI ≥ 25 kg/m^2^) increases the risk for LGA and fetal macrosomia (Table 2). In a sample of women from Canada, class 3 obesity (BMI ≥ 40 kg/m^2^, *n* = 249) was protective against LBW (aOR 0.07; 95% CI: 0.01, 0.36), but not SGA, compared to women with normal BMI (18.5–24.9 kg/m^2^, *n* = 446); but class 3 obesity was also associated with LGA (aOR 4.29; 95% CI: 2.67, 6.89); birthweight > 4000 g (aOR 3.70; 95% CI: 2.22, 6.16) and birthweight > 4500 g (aOR 5.78; 95% CI: 2.11, 15.86) [124]. In contrast, recent data from a retrospective 12-year cohort study of 75,432 women in Australia identified obesity as an independent risk factor for SGA compared to normal weight (adjusted OR 1.22; 95% CI: 1.14, 1.31) [130], similar to the 24% increased risk for SGA with maternal obesity in a multi-ethnic New Zealand population (adjusted OR 1.24; CI: 1.11, 1.39) [131].

**Table 2:** Odds ratios for adverse perinatal outcomes according to different pre-pregnancy/maternal body mass index.

Study Population	*n*	BMI (kg/m^2^)	Perinatal Outcome OR (95% CI)	
			LGA	SGA
Retrospective case-control study (*n* = 100), UK ^a^ [132]	Not reported	≥40.0	3.11 (1.25, 7.79) *	
Retrospective population-based cohort study of 5047 singleton nulliparous pregnancies, China ^b^ [133]	579	<18.5		1.67 (1.07, 2.61) ^†^
	926	24.0–27.9	1.46 (1.02, 2.08) ^†^	
	342	≥28	1.91 (1.17, 3.10) ^†^	
South Australian Pregnancy Outcome Unit, with singleton pregnancies (*n* = 19, 672) ^c^ [12]	364	<18.5	0.38 (0.22, 0.67)	2.12 (1.58, 2.85)
	2943	25.0–29.9	1.59 (1.41, 1.81)	0.75 (0.61, 0.92)
	1528	30.0–34.9	1.60 (1.37, 1.85)	0.77 (0.59, 0.99)
	684	35.0–39.9	1.91 (1.58, 2.30)	1.12 (0.82, 1.52)
	453	≥40.0	2.17 (1.76, 2.68)	0.56 (0.34, 0.94)
Birth cohort study. Queensland, Australia ^d^ [126]	211	≥30.0	2.73 (1.49, 5.01)	-
Singleton fetuses at the University of California (1981 through 2001). Weight measured on first pre-natal visit ^e^ [128]	Not reported	>29.0	3.04 (1.86, 4.98) White	-
			0.33 (0.04, 2.85) African American	-
			2.93 (1.00, 8.58) Latina	-
			3.55 (1.39, 9.07) Asian	-
Retrospective cohort study of women who had received prenatal care in the whole urban prenatal care centers of Kazerun, Iran ^f^ [129]	816	<19.8	0.48 (0.30, 0.77)	-
	682	26.0–29.9	1.27 (0.87, 1.86)	-
	186	≥29.0	1.21 (0.61, 2.41)	-
Prospective study in Thai women, at <28 weeks’ gestation ^g^ [125]	200	≥27.5	1.4 (0.5, 4.3)	-
Danish cohort of women carrying singleton births ^h^ [127]	116	<18.5	0.32 (0.27, 0.38)	-
	3160	25.0–29.9	1.70 (1.60, 1.78)	-
	1898	30.0–34.9	2.20 (2.08, 2.33)	-
	1363	≥35.0	2.73 (2.55, 2.94)	-
Retrospective cohort study among women and infants from the Better Outcomes Registry and Network dataset, Canada ^i^ [124]	249	≥40.0	3.70 (2.22, 6.16)	0.75 (0.38, 1.45)

* Fetal macrosomia (≥4000 g) ^†^ Relative Risk; ^a^ Reference BMI category: 20.0–25.0 kg/m^2^. ^b^ Analysis adjusted for maternal age, maternal education, and gestational weight gain. Reference BMI category: 18.5–24.9 kg/m^2^ (*n* = 3200). ^c^ Analysis adjusted for maternal age, parity, smoking status, and hospital status. Reference BMI category: 18.5–24.9 kg/m^2^ (*n* = 5,261). ^d^ Analysis adjusted for pre-pregnancy obesity, previous pregnancy, marital status, education level, and maternal smoking. Reference BMI category: <30 kg/m^2^. ^e^ Analysis adjusted for maternal age, parity, educational level, insurance status, gestational diabetes, gestational age, birthweight, induction of labor, use of epidural anesthesia, length of labor, and weight gain. Reference BMI category: 19.8–26.0 kg/m^2^. ^f^ Analysis adjusted for pre-pregnancy BMI and gestational weight gain, and additionally adjusted for maternal age, education level, occupation, family history of hypertension, family history of diabetes, and parity. Reference BMI category: 19.8–26.0 kg/m^2^. ^g^ Relative risk. Reference BMI category: 18.5–23.0 kg/m^2^. ^h^ Analysis adjusted for maternal age, parity, smoking during pregnancy, gestational age, birthweight, GDM, sex, and calendar year. BMI reference category: 18.5–24.0 kg/m^2^. ^i^ Analysis adjusted for maternal smoking, education quartile, and family income quartile. Reference BMI category: 18.5–24.9 kg/m^2^.

Several obesogenic models in animals have identified short-term consequences of maternal high fat feeding. In rats, exposure to maternal overnutrition or obesity during pregnancy was associated with obese offspring [134,135,136,137,138], with additive effects occurring when pups consumed a high fat diet post-weaning [139]. Overfeeding sheep from conception to mid-gestation to induce overweight and obesity also led to fetal overgrowth [140]. Other animal studies have investigated the long-term effects of excess maternal nutrition. One of the earliest was in the baboon, where overfeeding in the pre-weaning period permanently increased adiposity through larger fat cells; this was particularly evident in females [141]. Rats who were overfed to become obese prior to conception, were fed normally during pregnancy, and whose offspring were fed a normal diet, were more frequently obese as adults compared to controls [138]. Longer term high-fat feeding in rats also has been associated with insufficient placental function, reduced pup survival, and fetal death [142], suggesting that uterine stress as a result of maternal obesity alters proper development of the placental vasculature, leading to poor fetal development.

Obesity and pregnancy also individually contribute to a state of chronic inflammation. Maternal high fat consumption programs the appetite-regulating system of offspring towards orexigenic pathways, leptin resistance, and adiposity [33]. Mechanisms underpinning the relationship between maternal obesity and adverse perinatal outcomes have been reported; however, a biologic pathway has not been established. Pro-inflammatory cytokines are elevated in obese compared to normal pregnancies [143], which can ultimately impact the maternal–fetal interface, leading to a higher risk of fetal infection and fetal complications [144]. Maternal obesity also can result in placental oxidative stress [145]. The increase in oxidative stress may impair placentation and establishment of sufficient blood supply, potentially impacting fetal metabolism [146]. The placenta is involved with the delivery of nutrients, oxygen, and hormones to the fetus, thus is ultimately responsible for the growth trajectory of the fetus [147].

Increasing pre-pregnancy BMI has been associated with increased placental weight and placental size, which were associated with adverse perinatal outcomes [148]; and maternal obesity was also associated with placenta insufficiency and increased placental lesions [149]. Moreover, IUGR is associated with placental insufficiency [150]. As maternal obesity can also be associated with undernutrition in the mother as well as the fetus (reviewed in [151]), neonatal consequences include IUGR but also altered epigenetic programming, which has been linked to later life disease in the offspring (reviewed in [152]). It is difficult to identify whether fetal programming with maternal overweight/obesity shares the same mechanisms of maternal undernutrition; the mechanisms underlying the way in which fetal systems cope with excess fuels during intrauterine development remain relatively unexplored. These findings provide evidence that excess BMI plays a significant role in altered *in utero* environment, which can have significant adverse effects on the fetus both in the short and longer term.

#### Gestational Weight Gain

The effects of maternal obesity on maternal and infant outcomes are further confounded by pre-pregnancy BMI [153,154] as well as excess gestational weight gain [155,156,157,158]. In two recent systematic reviews and meta-analyses, high gestational weight gain was associated with lower risk of LBW (RR 0.64; 95% CI: 0.53, 0.78) [159], whereas low total gestational weight gain (<12.5 kg to <7 kg depending on pre-pregnancy BMI category) was associated with increased risk of LBW (RR 1.85; 95% CI: 1.72, 2.00) [160]. However, in a population-based cohort study of 3536 women with class 3 obesity, those who lost weight during pregnancy had increased risk for SGA babies (aOR 2.34; 95% CI: 1.15, 4.76) [161]. Subsequent meta-analysis on interventions aimed at reducing gestational weight gain revealed there were no significant differences in birthweight between the dietary/physical activity intervention and control groups [162,163]. The IOM has recently revised gestational weight gain recommendations according to different BMI categories which attempt to improve the short- and long-term health of the mother and the child [164].

### 3.6. Summary

The effects of maternal BMI on infant birthweight are important given the increasing prevalence of overweight and obesity in women of childbearing age. The strong evidence of association between childhood obesity and other metabolic abnormalities such as hypertension, diabetes, and cardiovascular disease signifies the importance of modifying body weight to an appropriate level prior to conception, to improve infant and adult outcomes. Although this is ideal, it is far from practical and is unlikely in a real-world situation. Dietary modification should be at the forefront of efforts to improve maternal health, regardless of BMI, in an effort to positively impact perinatal health. Further larger and more robust studies are required to identify the importance of limiting weight gain during pregnancy, and to follow up on longer term health outcomes in the mother, infant, and child.

## 4. Conclusions

This review summarizes comprehensive data from epidemiological studies and the latest evidence from RCTs on maternal dietary intakes and infant birthweight. Research to date has not identified an effective supplementation strategy to combat the adverse perinatal effects associated with suboptimal maternal diet; indeed, supplementation strategies may only be beneficial when a deficiency is observed. Thus, a complete understanding of the underlying dietary profile is warranted to avoid needless supplementation where no deficiency exists. In many cases, the mean birthweight of the study population was >3000 g, suggesting that any increases in birthweight that were identified may not be of clinical benefit. Suboptimal dietary intakes (intakes below recommended levels) occur not only in developing countries, but also in developed countries, irrespective of BMI. The global obesity epidemic brings new challenges in understanding, managing, and treating obesity in pregnancy, to improve both short- and long-term child health outcomes. Successful weight gain modifications show promise in reducing the risk of LGA babies but do not appear to modify rates of SGA. To date, the most promising results come from dietary pattern analyses, in which consumption of whole foods including fruit, vegetables, whole grains, low-fat dairy, and lean meats might be beneficial toward producing an infant of appropriate birthweight.

This review has highlighted the inconsistencies between studies and the limited success of dietary interventions studies to improve infant outcomes. We have identified the need for further RCTs that are carefully implemented and targeted to women with clear dietary deficiencies, as well as the need to reduce the increasing prevalence of women entering pregnancy with an overweight BMI through dietary interventions. Understanding pre-conception diet also deserves attention as dietary intakes appear to change minimally prior to and during pregnancy [165,166]. Up to 50% of pregnancies are unplanned [167]; therefore, informing women prior to pregnancy on the importance of healthy eating should be encouraged so as to maintain a healthy diet during pregnancy.
